# The cellulose synthase superfamily in fully sequenced plants and algae

**DOI:** 10.1186/1471-2229-9-99

**Published:** 2009-07-31

**Authors:** Yanbin Yin, Jinling Huang, Ying Xu

**Affiliations:** 1 Computational System Biology Lab, Department of Biochemistry and Molecular Biology, Institute of Bioinformatics, University of Georgia, BioEnergy Science Center, USA; 2 Department of Biology, Howell Science Complex, East Carolina University, Greenville, NC 27858, USA; 3 College of Computer Science and Technology, Jilin University, Changchun, Jilin, PR China

## Abstract

**Background:**

The cellulose synthase superfamily has been classified into nine cellulose synthase-like (Csl) families and one cellulose synthase (CesA) family. The Csl families have been proposed to be involved in the synthesis of the backbones of hemicelluloses of plant cell walls. With 17 plant and algal genomes fully sequenced, we sought to conduct a genome-wide and systematic investigation of this superfamily through in-depth phylogenetic analyses.

**Results:**

A single-copy gene is found in the six chlorophyte green algae, which is most closely related to the CslA and CslC families that are present in the seven land plants investigated in our analyses. Six proteins from poplar, grape and sorghum form a distinct family (CslJ), providing further support for the conclusions from two recent studies. CslB/E/G/H/J families have evolved significantly more rapidly than their widely distributed relatives, and tend to have intragenomic duplications, in particular in the grape genome.

**Conclusion:**

Our data suggest that the CslA and CslC families originated through an ancient gene duplication event in land plants. We speculate that the single-copy Csl gene in green algae may encode a mannan synthase. We confirm that the rest of the Csl families have a different evolutionary origin than CslA and CslC, and have proposed a model for the divergence order among them. Our study provides new insights about the evolution of this important gene family in plants.

## Background

The first plant gene encoding a cellulose synthase catalytic subunit (CesA) was identified in 1996 in cotton based on its sequence similarity to a bacterial CesA [1]. In 2000, Richmond and Somerville identified 10 CesA genes and 31 cellulose synthases-like (Csl) genes in Arabidopsis, which were further classified into one CesA family and six Csl families (CslA/B/C/D/E/G) based on phylogenetic analyses [2]. Since then, the whole CesA and Csl gene repertoire has been cataloged in fully sequenced plants, including rice [3], poplar [4,5] and the moss *Physcomitrella patens *[6]. Additional CesA and Csl genes have also been found in diverse and not fully sequenced land plants such as maize [7], barley [8] and pine [9]; CesAs have been identified in streptophyte green algae such as *Mesotaenium caldariorum *[10,11] and in red alga *Porphyra yezoensis *[12,13] as well. Two additional Csl families (CslF and CslH) were found in these studies; together with the other six Csl families and one CesA family, they comprise the CesA superfamily.

The CesA superfamily genes are among the most important players involved in the biosynthesis of plant cell walls, which are mainly composed of biopolymers such as celluloses, hemicelluloses, pectins and lignins. Because the Csl genes share sequence similarities with the CesA genes, they are hypothesized to be involved in the biosynthesis of the backbone of various polysaccharide polymers [2], in particular hemicelluloses [14]. This so-called "CSL hypothesis" has been supported by recent experimental studies, which suggest that the CslA genes encode the mannan synthases [15,16], the CslF and CslH genes encode the mixed linkage glucan synthases [17,18], and the CslC genes are probably involved in the xyloglucan biosynthesis [19]. Therefore the backbone synthases of all major hemicellulose classes except for xylans are known. However, the functional roles of the other Csl families (CslB/D/E/G) remain unclear.

The phylogenetic classification and the function of the CesA superfamily were reviewed by Lerouxel *et al*. in 2006 [14], and since then there have been a few updates in terms of the phylogenetic analyses of these important genes. Fincher *et al*. have found a new Csl family (CslJ) in cereals [20,21]. Roberts and Bushoven have mined the *P. pattens *genomic and EST data and found CesA, CslA, CslC and CslD genes in this lower plant [6]; their phylogenetic analyses revealed that seven *P. patens *CesA genes form a monophyletic clade by themselves and there are no one-to-one orthologs in the moss corresponding to the Arabidopsis CesA triplet subunits (CesA1/3/6 for the primary cell wall and CesA4/7/8 for the secondary cell wall). Furthermore, comprehensive phylogenetic analyses of the plant CesA superfamily by including CesAs from other organismal groups (e.g., bacteria, fungi and animals) indicated that plant CslA and CslC genes have a different origin than the remaining plant genes [22]. Evidences have been reported that these remaining genes of the CesA superfamily were anciently acquired from cyanobacteria [23]. It was proposed [22] that the plant CslG genes evolved first, followed by the CslE, CslB, CesA and CslD/F genes. However, a more recent study could not find homologs of the CslG/E/B/H/F genes in *P. patens *[6], suggesting that these Csl families are narrowly distributed and unlikely to be the earliest evolved.

To date 17 plant and algal genomes have been fully or nearly fully sequenced, and their gene prediction and annotation are publicly available (Table 1). The availability of these genomes and their annotated genes facilitates comparative genomic studies of plants, making it possible to address major plant biology questions *in silico *[24]. We have performed comparative analyses of the CesA superfamily genes in the 17 sequenced plant and algal genomes. Our goals are to define CesA and Csl gene homologs across these genomes and to investigate the evolution of different Csl gene families. We have built a catalog of all the Csl genes and classified them phylogenetically. The gene structure, the evolutionary rate, and the distribution of the Csl families across different genomes are also studied. Throughout this paper, we use Csl genes to denote all cellulose synthases-like genes including CesAs.

**Table 1:** Plant and algal genomes used in the present study

Index	Abbr.	Clade	Species	Genome Published/Released	Csl Published?
1	Tp	Diatom	*Thalassiosira pseudonana*	[25]	N
2	Pht	Diatom	*Phaeodactylum tricornutum*	JGI	N
3	Pa	brown tide algae	*Aureococcus anophagefferens*	JGI	N
4	Cm	red algae	*Cyanidioschyzon merolae*	[26]	N
5	Mpc	green algae	*Micromonas pusilla CCMP1545*	[27]	N
6	Mpr	green algae	*Micromonas strain RCC299*	[27]	N
7	Ol	green algae	*Ostreococcus lucimarinus*	[28]	N
8	Ot	green algae	*Ostreococcus tauri*	[29]	N
9	Cr	green algae	*Chlamydomonas reinhardtii*	[30]	N
10	Vc	green algae	*Volvox carteri f. nagariensis*	JGI	N
11	Pp	moss	*Physcomitrella patens ssp patens*	[31]	[6]
12	Sm	spike moss	*Selaginella moellendorffii*	JGI	N [32]
13	Pt	dicot	*Populus trichocarpa*	[33]	[5]
14	At	dicot	*Arabidopsis thaliana*	[34]	[2]
15	Vv	dicot	*Vitis vinifera*	[35]	N
16	Os	monocot	*Oryza sativa*	[36,37]	[3]
17	Sb	monocot	*Sorghum bicolor*	[38]	N

## Results

### Identification of Csl proteins

We analyzed the Csl genes in the 17 genomes based on BLAST [39] and HMMER [40] searches (see Methods for details). We found that all predicted Csl proteins contain either the Pfam Cellulose_synt domain (PF03552, 898 aa long) or the glycosyltransferase family 2 (GT2) domain (PF00535, 149 aa long) but not both. For example, all CslA and CslC proteins in Arabidopsis have the GT2 domain but not the Cellulose_synt domain, while all other Arabidopsis Csl proteins contain only the Cellulose_synt domain, which is consistent with a previous finding that CslA and CslC have a different origin than the other Csl families [22]. We then grouped all proteins with the GT2 domain into a set denoted as the GT2 dataset, and those with the Cellulose_synt domain into the Cellulose_synt dataset. These two datasets do not share any common proteins.

By querying the Pfam Cellulose_synt domain, we identified two cyanobacterial proteins from the fully sequenced bacterial genomes. These two proteins were originally identified by Nobles *et al*. in 2001 [23], and here are used to root the eukaryotic Cellulose_synt phylogeny.

### Phylogenetic classification of Csl families

**Figure 1:**
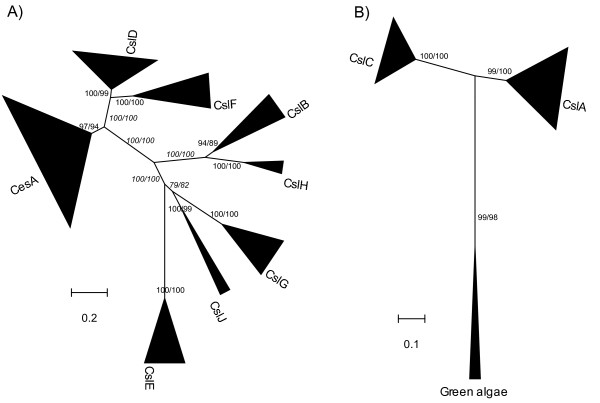
**The maximum likelihood (ML) phylogenies of the Csl families**. a) 217 plant proteins (211 proteins if alternatively splicing variants from Arabidopsis and rice are excluded) that have the Pfam Cellulose_synt domain were used to construct this tree. b) 88 proteins (83 proteins if alternatively splicing variants are excluded) that have the Pfam GT2 domain were used to construct the tree. Both the full length protein sequences and the conserved Pfam domain regions were used in the phylogeny reconstruction and the corresponding bootstrap values are shown and split by '/'.

To characterize the identified Csl genes from the 17 genomes, we built phylogenetic trees for each of the two aforementioned datasets, based on the multiple sequence alignments of both the full length proteins and the conserved Pfam domains (see Methods for details). Figures 1A and 1B show the un-rooted maximum likelihood (ML) trees for the Cellulose_synt dataset and the GT2 dataset, respectively. The number and the species information of the genes included in the phylogeny are given in Table 2. Neighbor joining (NJ) trees for the two datasets were also constructed and given in the Additional file 1 [see Additional file 1].

**Table 2:** Sizes (number of genes in each family) of Csl families in 17 plant and algal genomes

Abbr. ^a)^	GenomeSize^b)^	Sum.	CesA	CslD	CslF	CslB	CslH	CslE	CslG	CslJ	CslA	CslC
Tp	11390	-	-	-	-	-	-	-	-	-	-	-
Pht	10025	-	-	-	-	-	-	-	-	-	-	-
Aa	11501	-	-	-	-	-	-	-	-	-	-	-
Cm	5014	-	-	-	-	-	-	-	-	-	-	-
Mpc	10475	1	-	-	-	-	-	-	-	-	1	
Mpr	9815	1	-	-	-	-	-	-	-	-	1	
Ol	7651	1	-	-	-	-	-	-	-	-	1	
Ot	7725	1	-	-	-	-	-	-	-	-	1	
Cr	14598	1	-	-	-	-	-	-	-	-	1	
Vc	15544	1	-	-	-	-	-	-	-	-	1	
Pp	35938	26	8	8	-	-	-	-	-	-	3	7
Sm	34697	22	10	6	-	-	-	-	-	-	2	4
Pt	58036	50	18	11	-	2	-	3	4	2	5	5
At	31921	39^c)^	10	6	-	6	-	1	3	-	8	5
Vv	30434	58	11	5	-	7	-	9	15	3	4	4
Os	66710	44	10	5	8	-	2	3	-	-	10	6
Sb	35899	49	12	5	11	-	3	3	-	1	8	6
Total		294	79	46	19	15	5	19	22	6	45	37

All		294	211								83	

^a) ^See Table 1 for species full names

^b) ^Genome size is measured as the number of protein coding genes in each genome.

^c) ^AtCslA1 was not identified in our hmmsearch (see Methods for details), and thus the number of Csl genes in Arabidopsis is shown to be 39 instead of 40.

The number of proteins in each Csl family, calculated based on their groupings as shown in Figure 1, is given in Table 2. No Csl genes were detected in the three stramenopile algae (two diatoms: *Thalassiosira pseudonana*, *Phaeodactylum tricornutum *and one brown tide alga: *Aureococcus anophagefferens*) and the red alga *Cyanidioschyzon merolae*. Only one single-copy gene, which is most closely related to CslA/C, was identified in each of the six green algae. Overall, 77 proteins from land plants have the GT2 Pfam domain, which are assigned to either the CslA or the CslC family (Figure 1b). Six additional proteins from green algae also have the GT2 Pfam domain and are the most homologous to both the land plant CslA and CslC genes. The Cellulose_synt Pfam domain is found in 211 proteins, which are assigned to the other Csl families (Figure 1a). Based on their taxonomic distribution and the phylogenies shown in Figure 1, we have divided the 294 identified Csl proteins into three categories. The first category consists of CslA and CslC, the most conserved Csl families in our analyses. These families are found in all seven land plants and all six green algae sampled in this study. The second category contains three phylogenetically related families, CesA, CslD and CslF (Figure 1a). Among these families, CslF is specific to grasses while CesA and CslD are present across all the sampled land plants, including the two lower plants (*P. patens *of bryophytes and *S. moellendorffii *of lycophytes in Table 1). The last category includes the remaining Csl families, which appear to be confined to the five sampled seed plants (Table 2). Such a restricted distribution suggests a likely late origin of these Csl families and possible functional roles specific to seed plants.

### CslJ represents a new Csl family that is found in both monocot and dicot plants

**Figure 2:**
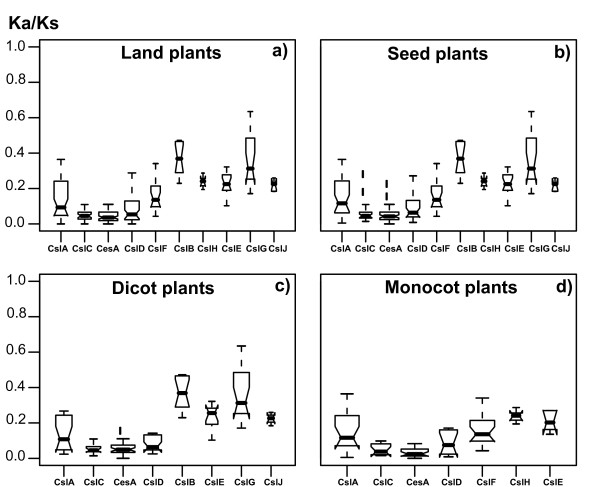
**Estimation of evolutionary rates of different Csl families**. If the Ka/Ks ratio for a protein is less than one (less amino acid replacements than silent base substitutions), it means the protein is under negative selection; otherwise if Ka/Ks>1, it means the protein is under positive selection. We used the model = 1 implemented in codeml of PAML to compute Ka/Ks, which allows each gene in the tree evolving at its own rate; therefore within each family (tree) a different Ka/Ks value for each gene is obtained (see Methods for details). The distribution of the Ka/Ks values of genes of each Csl family is plotted side by side, showing the minimum, the 25% percentile, the median, the 75% percentile and the maximum values of Ka/Ks. The boxes are drawn with widths proportional to the square-roots of the number of genes in the groups. A notch is drawn on each side of the box towards the median. For panel a) we used Csl genes from all the seven land plants; for b), c) and d) we extracted and plotted genes from only subsets of the seven genomes, namely, the five seed plants, the three dicot plants and the two monocot plants, respectively.

Figure 1 shows all the nine previously known Csl families [14], namely CesA and CslA/B/C/D/E/F/G/H, each forming a well-supported group. A new Csl family was discovered very recently in cereals and named CslJ [20,21]. Here we found this family not only in sorghum but also in poplar and grape, suggesting that it is another seed plant-specific Csl family in addition to the CslE family. The sorghum sequence (Sb03g047220) was found in both this study and previous papers [20,21]. According to the phylogeny (Figure 1a; [see Additional file 1]), CslJ is closely related to CslG. We have examined if the CslJ family contains functional genes, knowing that neither Arabidopsis nor rice contains this family (Table 2). The following evidence suggests that genes of this family are not likely to be pseudogenes. First, our Ka/Ks analysis (see Methods for details) shows that members of this family are under negative selection (median of Ka/Ks = 0.23, Figure 2). Second, all members of this family (Table 3) have EST sequences in the NCBI EST database, indicating that these genes are expressed. And third, members of this gene family have also been found in unfinished genomes such as barley, wheat and maize [20,21].

**Table 3:** Expression of members of the CslJ family

CslJ gene ID	NCBI accession numbers of ESTs	UniGene	Tissue/Organ
fgenesh1_pg.C_LG_X000708	DB888819.1, CV257302.1, DB906752.1		Mixture of leaf, bud, stem, root
estExt_fgenesh1_pg_v1.C_LG_X0702	DB885869.1, DN497067.1, BU871140.1, AJ772607.1, DN487448.1, AJ770380.1, DB903904.1, AJ772118.1		Dormant bud, mixture of leaf, bud, stem, root
Sb03g047220	CF430961.1, CF431079.1		Nitrogen-deficient seedlings
GSVIVP00020164001	CF211163.1, EE094868.1, EE097022.1, EC990611.1, CV100631.1, CF983720.1, EE086006.1, EC925887.1, DT021105.1, EC927377.1, CF211254.1, DT010825.1, CF210160.1, CN006709.1, CF515516.1, CF515427.1, CF210083.1, EE093327.1, EE093253.1, CV179236.1	Vvi.14469	Fruit; flower; leaf; mixed; cell culture
GSVIVP00020168001	FC063595.1, EC948646.1, DT004980.1	Vvi.20726	Flower, leaf and root
GSVIVP00020169001	EE094198.1, CF983803.1, EE100185.1		Leaf and berry

Protein sequences of the CslJ members (first column) were queried to search against the NCBI EST database. With E-value cutoff <= 1e-2, EST matches that are >98% identical to the query protein and are from the same species as the query were collected and listed in the second column. These ESTs matches were manually checked at the NCBI website to find UniGene links (third column) and tissue/organ expression information (fourth column) if they have.

### A green algae-specific family

Six green algal Csl genes (each green alga contains a single-copy of this gene; see Table 2) form a well-supported group distinct from the land plant CslA and CslC genes (Figure 1b). Inasmuch this green algal Csl group possibly represents the most homologous genes of the common ancestor of CslA and CslC in all the land plants.

### Evolutionary rate of Csl families

The ratio between Ka and Ks (see Methods for details) has been widely used to measure the selection pressure on proteins [41]. Generally, a lower selection pressure indicates a higher evolutionary rate. Using Ka, Ks and the Ka/Ks ratio as proxies, people have found significant correlations between protein evolutionary rates and numerous features derivable from genome sequences, such as the number of protein-protein interaction partners, gene expression levels, the essentiality of a gene and the number of paralogs of a gene [42-44] (and papers cited therein). In particular, genes that originated relatively recently through gene duplications or by other mechanisms usually evolve more rapidly than the more ancient genes [45]. In order to compare the relative evolutionary rates among the Csl families that may have come into being through duplications [46], we have calculated the Ka/Ks ratios (see Methods for details) and conducted rigorous statistical analyses on the computational results.

The comparisons of the distribution of the Ka/Ks ratios across different Csl families are shown in Figure 2a–d, from which it is clear that the narrowly distributed Csl families (CslB/H/E/G/J) tend to have higher Ka/Ks ratios than those widely distributed Csl families (CslA/C/D and CesA). This observation is statistically supported by pair-wise Wilcoxon tests (Table 4a–d). Interestingly, this observation remains to be true when we compared the genes of the Csl families across different groupings of the plant genomes, namely across (a) all the seven land plants (Figure 2a), (b) the five seed plants (excluding moss and spike moss from (a); Figure 2b), (c) the three dicot plants (Figure 2c), (d) the two monocot plants (Figure 2d), and (e) each of the seven plants (data not shown). Specifically, we found that the dicot-specific CslB and CslG families have evolved the most rapidly, followed by the monocot-specific CslH, and then by the seed plant-specific CslE and CslJ. In addition, the monocot-specific CslF has evolved significantly more rapidly than CslC and CesA, but not than CslA and CslD (Table 4d). Overall, we found that the CslB/H/E/G/J families have evolved more rapidly than the other Csl families, which lends further support for the hypothesis that these families might have diversified relatively recently to acquire new functions specific to the seed plants.

**Table 4:** Two sample (pair-wise) nonparametric Wilcoxon test P values (Csl family in the column vs. in the row)

All plants (a)	CslA	CslC	cesA	CslD	Seed plants (b)	CslA	CslC	cesA	CslD
	
CslF	0.1584*	2.76e-05	7.44e-07	0.0009	CslF	0.2707*	5.66e-06	1.26e-07	0.0065
CslB	3.36e-05	1.10e-06	5.56e-08	2.05e-06	CslB	3.52e-05	4.93e-10	1.78e-08	3.93e-08
CslH	0.0307	0.0012	0.0006	0.0033	CslH	0.0349	7.06e-05	0.0002	0.0020
CslE	0.0017	8.94e-07	3.64e-08	6.22e-06	CslE	0.0028	7.27e-09	3.43e-09	3.46e-06
CslG	3.97e-05	1.84e-07	2.94e-09	3.34e-07	CslG	5.26e-05	1.07e-10	5.71e-10	1.21e-08
CslJ	0.2187*	0.0261	0.0156	0.0463	CslJ	0.2554*	0.0178	0.0010	0.0805*
	
									
Dicot plants (c)	CslA	CslC	cesA	CslD	Monocot plants (d)	CslA	CslC	cesA	CslD
	
CslB	0.0010	4.99e-08	4.97e-08	3.23e-08	CslF	0.3520*	0.0008	8.69e-06	0.0521*
CslE	0.0185	2.04e-06	9.67e-07	0.0001	CslH	0.0399	0.0019	0.0013	0.0063
CslG	0.0017	3.77e-09	2.13e-09	3.18e-08	CslE	0.0386	0.0024	0.0007	0.0112
CslJ	0.0933*	0.0003	0.0006	0.0123					

In a hypothesis testing, a P-value is usually calculated to indicate if the null hypothesis is statistically supported. In this case, genes of Csl families in the column are tested against those in the row in terms of their Ka/Ks values. The null hypothesis is that the column is equal to the row, and the alternative hypothesis is that the column is larger than the row. If the P-value is less than 0.05, the null hypothesis is rejected and the alternative hypothesis is statistically significantly supported. This table shows that except for a very few cases (those with asterisks), the columns are always significantly larger than the rows.

### Comparative study of individual Csl families

We have compared the Csl genes across different plant genomes by inspecting the phylogeny of each family and analyzing the gene structures. We present here a detailed comparative analysis of the less studied and narrowly distributed CslB/H/E/G/J families. The analyses of the other Csl families are given in the Additional file 2 [see Additional file 2].

#### A) CslE family

**Figure 3:**
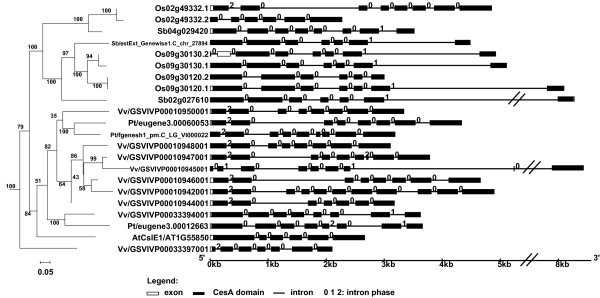
**The subtree of the CslE family and gene structures of the family members**. This ML phylogeny is taken from Figure 1a. The gene structure was plotted using the GSDS server [71]. The branch length is scaled, i.e. proportional to the estimated number of molecular change. A scale bar is shown under the tree. The bootstrap values are shown to indicate the confidence level of the grouping. The intron-exon structure is shown on the right. The intron phase indicates the position of the intron within a codon. If it is not located within a codon (or located between two codons), the phase is 0. If it is located within a codon (or split a codon into two exons) and after the first base of the codon, the phase is 1, otherwise the phase is 2.

The phylogeny and the gene structure of the CslE family are shown in Figure 3. While there is only one CslE gene in Arabidopsis, nine copies of the gene are found in the recently sequenced grape genome (Table 1). Six of these grape genes form a monophyletic group, and are located on the chromosome in tandem within a ~45 kb region, which apparently resulted from a tandem duplication event. All these grape genes have Ka/Ks ratios between 0.24~0.33 (under purifying selection) and have EST data in GenBank, suggesting that they are functionally active. The grass genes form a large group on the phylogeny, suggesting that CslE diverged separately after the split of dicots and monocots.

#### B) CslG and CslJ families

**Figure 4:**
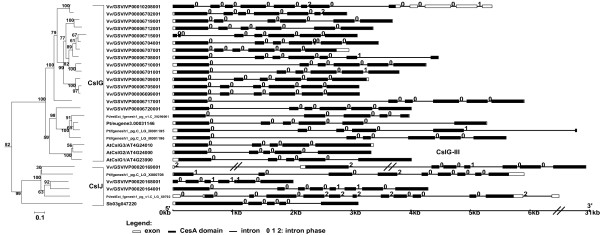
**The subtree of the CslG/J families and gene structures of the family members**. See the legend of Figure 3.

These two families are closely related to each other in the phylogeny shown in Figure 1. Genes of the dicot-specific CslG family [14] form mostly genome-specific monophyletic clusters (except for CslG-II that has a grape gene), and have conserved gene structures. This suggests that the CslG family diversified separately in each dicot plant via intragenomic duplications. Similar to CslE, this family is also substantially expanded in the grape genome possibly as a result of tandem gene duplications (Table 2 and Figure 4). The 14 grape CslG genes have Ka/Ks ratios between 0.20~0.65; nine of them have ESTs in GenBank, and one has more than ten identical ESTs (UniGene entry Vvi.9751). The EST data indicate that this gene is expressed mostly in grape leaves, although further experimental studies are clearly needed to determine if any of these duplicated genes is actually functional.CslJ is possibly a new family according to recent studies [20,21] and our current analyses. Although CslJ is closely related to CslG, these two families have very different gene structures (Figure 4). In addition, unlike other families, the CslJ genes appear to be less conserved in their gene structures.

#### C) CslB and CslH families

**Figure 5:**
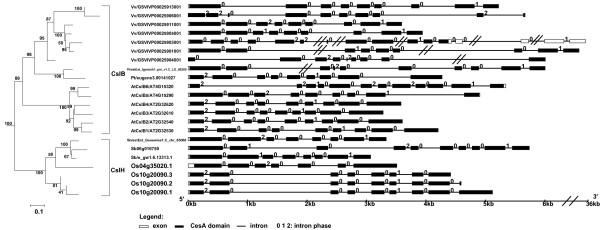
**The subtree of the CslB/H families and gene structures of the family members**. See the legend of Figure 3.

These two families are phylogenetically related as shown in Figure 1. CslB is a dicot-specific family while CslH is monocot-specific [14]. We have observed that the paralogous genes of these two families tend to form clusters and have similar gene structures (except for Os04g35020.1) (Figure 5), suggesting the possibility of independent genome-specific duplications followed by subsequent sequence divergence.

## Discussion

The cellulose synthase-like gene families are among the most important players involved in the formation of plant cell walls. Some Csl families have been found to be responsible for the synthesis of the backbone of hemicelluloses, while the others are yet to be functionally characterized [14]. This study focuses on the computational identification and classification of the Csl families in fully or nearly fully sequenced plant and algal genomes. Our phylogenetic analyses have led to the identification of the Csl orthologs across these genomes and the generation of new insights about how they might have evolved in plants and algae.

### Limitation in identifying CesAs forming linear terminal complexes (TCs)

Our analyses did not detect any CesA and Csl genes in the three stramenopile algal genomes nor in the red algal genome, but this does not necessarily mean that no stramenopiles or red algae contain these families. In fact, a CesA gene has been identified very recently in an yet sequenced red alga *Porphyra yezoensis *[13]. Actually, the cellulose synthases, which assemble into the so-called terminal complex (TC), have been found across all classes of organisms. TCs can be morphologically classified into rosette TCs or linear TCs [47-49], for each of which the component CesAs have rather different domain structures [48]. While the rosette TCs have been extensively studied and found in all seed plants, the linear TCs may be the most ancient, given their wider distribution across bacteria, fungi, animals and many classes of algae including stramenopiles [48,50].

Since the focus of this study is on CesAs that form rosette TCs, we have adopted a rather conservative filtration procedure (see Methods for details), which may have excluded some GT2 proteins that are possibly forming linear TCs. For instance, one GT2 gene from *P. patens *and seven GT2 genes from *S. moellendorfii *were removed by our filtration procedure (data not shown) but have been found to be similar to the CesA genes of cyanobacteria and the red alga *Porphyra yezoensis *[32].

### The green algal CslA/C-like genes and their possible functions

Despite all the previous studies about the distribution of CesAs, very little has been done to identify the other Csl families across different organisms. So what is known about these families is fragmented at the best. For example, a sequence fragment from *Chlamydomonas *was reported to have diverged early from the CslA and CslC families [22]. In our study, we found that all the six sampled chlorophyte green algae have a single-copy gene that is the most homologous to the land plant CslA and CslC families. Our phylogenetic analysis indicates that the CslA and the CslC families might have evolved via a gene duplication event that occurred uniquely in land plants after they split from green algae (Figure 1b).

CslAs and CslCs have been characterized to encode mannan synthases [15,16] and xyloglucan synthases [19], respectively. It is tempting to speculate that the mannan synthesis might represent the ancestral function for the single-copy CslA/C-like genes in green algae, since mannan is present in both charophyte algae and chlorophyte algae while xyloglucan is absent from these green algae [51,52].

### The divergence order of Csl families

Recent studies have shown that the plant CesA genes and some other Csl families are likely of cyanobacterial origin [22,23], possibly as a result of intracellular gene transfer from plastids (or cyanobacterial endosymbionts). In a Csl gene phylogeny built by Nobles and Brown, CslG was suggested to be the first Csl family evolved in plants, followed by CslE, CslB, CesA and CslD [22]. However, our search of fully sequenced plant and algal genomes has shown that CslG is among the most narrowly distributed Csl families, and is absent from our sampled algae and lower plants (Table 2). Intuitively, such a distribution pattern suggests that CslG might not be the earliest plant Csl family.

**Figure 6:**
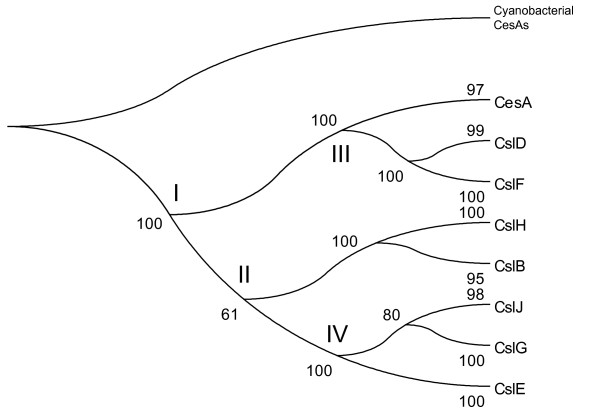
**A phylogeny of the Csl families based on the multiple sequence alignment of 219 full-length protein sequences**. 217 proteins were used in Figure 1a. Two cyanobacterial sequences (YP_322086.1 from *Anabaena variabilis ATCC 29413 *and NP_487797.1 from *Nostoc sp. PCC 7120*) were used as the out-group to root the phylogeny.

When constructing the phylogeny of Csl families (Figure 6), we included two cyanobacterial CesA protein sequences (YP_322086.1 from *Anabaena variabilis ATCC 29413 *and NP_487797.1 from *Nostoc sp. PCC 7120*) as the out-group of the plant CesA and Csl genes. These sequences are the only two hits (see the first section of Results) in our hmmsearch with the Pfam Cellulose_synt domain against all fully sequenced prokaryotic genomes (with E-value cutoff < 1.0). These two cyanobacterial sequences are phylogenetically more closely related to the land plant rosette-TC-forming CesAs than to the other bacterial linear-TC-forming CesAs [22], and thus have been proposed as the progenitor of all the rosette-TC-forming CesAs and Csl genes. The rooted Csl phylogeny (Figure 6) suggests a major early split between CesA/CslD/F and the other Csl families (at node I). Clearly neither CslG nor any of the other Csl families could be considered as the earliest in plants, because the cyanobacterial CesAs are not placed closer to any particular plant Csl family than to the others. Instead, the Csl phylogeny suggests that, after the establishment of plastids (or cyanobacterial endosymbionts) in the ancestral plant, the cyanobacterial derived CesA gene might have undergone several rounds of sequence and function divergence (see nodes I to IV in Figure 6). The evolutionary relationship of CslB/H to the other families is less clear from our phylogeny since the grouping of CslB/H with CslE/G/J has only a modest support (61% bootstrap value).

The finding that CslB/H/E/G/J split earlier than CslD/F (I *vs*. III) is surprising, because CslD is found in lower plants (moss and spike moss) whereas CslB/H/E/G/J are not. One plausible explanation is that some ancestral genes of the CslB/H/E/G/J families might have been present in the most recent common ancestor of the land plants, but were lost in moss and spike moss later. To test this hypothesis, we performed a TFASTY [53] search of the Csl proteins against the *P. patens *genomic sequences and the *S. moellendorffii *genomic sequences (Table 2), respectively, after masking out all predicted genes. Interestingly we found that some short sequence fragments are more similar to CslB/E/G than to the other Csl families, suggesting the possibility that they are the remnants of CslB/H/E/G/J. For example, the *P. patens *scaffold_21 region from position 2584724 to position 2584398 (in base pair) is 28% identical (51% similar) to the poplar CslB protein eugene3.00141027 from position 7 to position 109 (in amino acid), whose Cellulose_synt pfam domain is from position 9 to position 747; and the *S. moellendorffii *scaffold_577 region from position 192 to position 741 is 27% identical (52% similar) to the rice CslE protein Os09g30120.1 from position 533 to position 708, whose Cellulose_synt pfam domain is from position 15 to position 737. Similar searches against green algal genomic DNAs also found some homologous regions to the land plant CslB/H/E/G/J genes.

### The seed plant-specific CslB/H/E/G/J families

Although the common ancestor of the CslB/H/E/G/J families may have split from the other Csl families during the early evolution of plants, their diversification might have occurred recently. This is supported by the narrow distribution of CslB and CslG in dicots and CslH in monocots. Additionally, we have shown that all these five families have evolved rapidly (Figure 2). Paralogous genes of these families typically form a monophyletic group in our phylogenies and have highly similar gene structures (Figure 3, 4, 5). These findings suggest that recent intragenomic duplications have played a major role in the rapid sequence and functional diversification of these families [46]. A simple TBLASTN [39] search of the Csl proteins against the masked plant genomic DNA sequences (with all annotated gene models masked) found many homologous DNA fragments, indicating that they are likely pseudogenic relics after gene or genome duplications. A detailed analysis of these DNA fragments could possibly lead to a deeper understanding about the evolution of these Csl families.

## Conclusion

The cellulose synthase superfamily is identified and phylogenetically analyzed in fully sequenced plant and algal genomes. We conclude that 1) a CslJ family is present in both monocot and dicot plants, confirming two previous reports that it is a new Csl family, and that 2) a green algae-specific Csl family is most homologous to both land plant CslA and CslC families and it is speculated to be a mannan synthase, and that 3) CslG may not be the first evolved Csl family and a new model is proposed in regard to the evolution order of different Csl families.

## Methods

### Data sources

We downloaded the genome, proteome, and gene prediction and annotation data for the 17 genomes from various sources (Table 1). Specifically, the Arabidopsis data were from The Arabidopsis Information Resource (TAIR version 7.0) [54], rice data from The Institute for Genomic Research (TIGR version 5.0) [55], grape data from [56], red algae data from [57], and data for all the others are from Joint Genome Institute (JGI) as of Dec. 2007. The Arabidopsis and rice proteome data include alternatively splicing variants, while all the other proteomes do not. We included these splicing variants of the two organisms in our analyses, but counted them as one gene in our statistics in Table 2. For instance, AtCslA3 has three known splicing variants (AT1G23480.1, AT1G23480.2 and AT1G23480.3); all these three proteins were included in our phylogenetic analyses, although we count them as one single gene in Table 2. We specified these variants in our gene structure figures such as Figures 3, 4, 5. In addition, 597 fully sequenced prokaryotic genomes were downloaded from [58] as of Dec. 2007.

### BLAST search

We downloaded known Csl sequences from the Cell Wall Navigator database [59,60], including all known Arabidopsis and rice Csl genes as well as sequences from other species in UniProt [61]. We took this data set as the initial query to search against the annotated protein sequences of the 17 genomes.

### HMMER search

There are two Pfam [40] domain models for the Csl proteins: PF03552 (Cellulose_synt) and PF00535 (Glycos_transf_2 or GT2), both of which were searched in our analyses. In addition, we searched bacterial cellulose catalytic domain model PF03170 (BcsB) but did not find any significant hits (with E-value cutoff < 1.0) in plants and algae. We ran hmmsearch against proteins of the 17 genomes by querying these HMM models in the ls mode (global with respect to query domain and local with respect to hit protein; see details in the manual of HMMER package). We also performed hmmsearch against the 597 prokaryotic genomes.

### Selection of homologs

We have processed the search hits obtained from the above BLAST and HMMER searches in order to build an accurate Csl gene catalog for each genome:

#### a) Intersection of search results

We removed all the BLAST and hmmsearch hits with E-value higher than 1.0, and kept only the hits returned by both BLAST and hmmsearch; that is, the final hits should be similar to the query Csl genes in the pair-wise sequence comparison and contain either of the two conserved Pfam domains. We used E-value cutoff < 1.0 for both BLAST and hmmsearch because, under this condition, all known Arabidopsis Csl proteins except for AtCslA1/AT4G16590 (therefore in Table 2 the number of Arabidopsis Csl genes is 39) were successfully retrieved and no false positives were found. In addition, under this condition, the best Arabidopsis homologs of all the identified Csl sequences are known Csl genes.

#### b) Further filtration

We searched the candidate Csl proteins against the 17 genomes. For the true Csl genes, we expect to see their top hits in the candidate Csl gene list. For each candidate, we manually inspected its top 10 non-self hits: a candidate was dropped if fewer than eight of the top 10 non-self hits were in the candidate list. A few additional sequences were found to be not Csl genes in the subsequent more rigorous phylogenetic analyses and were removed. The FASTA format sequences of all the finally identified CesA and Csl genes are given in the Additional file 3 [see Additional file 3].

### Phylogenetic analysis

Two datasets were prepared for our phylogenetic analysis: protein sequences that contain the PF00535 (GT2) domain and those that have the PF03552 (Cellulose_synt) domain. Multiple protein sequence alignments (MSAs) were performed on both the full length regions and the conserved Pfam domains for the two datasets. MAFFT [62] was used in these alignments using two highly accurate methods: L-INS-i and E-INS-i. L-INS-i is considered to be the most accurate MSA method [63,64], and E-INS-i performs well on sequences with large unalignable regions (see manual of MAFFT). The resulting MSAs were manually edited to remove gaps and ambiguously aligned regions. We have also inspected the MSAs for the presence of the DXD and D, D, D, QXXRW motifs that are characteristic of possessive β-glycosyltransferases [65]. The original MSAs, the edited MSAs and the resulting phylogenetic trees are all available in the Additional file 4 [see Additional file 4].

The ProtTest v1.4 package [66] was run on the computed MSAs to select the best-fit models for phylogenetic analyses. We found the combination of JTT+I+G+F models to be the best one for our phylogeny reconstruction. The maximum likelihood (ML) trees were built using PhyML [67], while neighbor-joining (NJ) trees were built using MEGA4 [68] considering the above models. Specifically, PhyML analyses were conducted using the JTT model, 100 replicates of bootstraps, an estimated proportion of the invariable sites (I), four rate categories, an estimated gamma distribution parameter (G), and optimized starting BIONJ tree. MEGA analyses were conducted using the JTT substitution model, 500 replicates of bootstrap, pair-wise detection of gaps or missing data, gamma distributed rate among sites and the gamma parameter set at 1.0 (G).

### Evolutionary rate computation

The evolutionary rate of proteins can be estimated by calculating the evolutionary distances that are often measured by calculating the ratio of the number of nonsynonymous substitutions per nonsynonymous site (Ka) and the number of synonymous substitutions per synonymous site (Ks) [43]. For each Csl family, in order to obtain the longest possible alignment, we firstly built an initial multiple sequence alignment on the full length Csl protein sequences, and then manually examined the alignment to remove fragmental sequences that introduced long gaps into the alignment (e.g. for those less well annotated genomes, the predicted protein sequences are often of low quality and fragmented). We then rebuilt the multiple sequence alignment on the remaining sequences and reconstructed an un-rooted ML tree.

We transformed the amino acid sequence alignment into codon sequence alignment by using pal2nal [69]. The coding sequences were obtained from the downloaded genome data. The maximum likelihood estimation of Ks, Ka and Ka/Ks values for each gene family was conducted by running codeml in PAML [70], using the above tree and the codon alignment as the input. The computation of Ka/Ks ratios for a group of genes based on their phylogeny is conducted under the assumption that each gene evolves at an independent rate. We used this model to compute the Ka/Ks ratio for each gene of each Csl family. Analyses were also performed using the conserved domain regions; the result as shown in Figure 2 remains unchanged (data not shown).

### Gene structure analysis

The gene structure information was parsed from the GFF file downloaded along with the genome data, and was used as the input for the graphic display at the Gene Structure Display Server of Peking University [71].

## Authors' contributions

YY conceived this study, conducted all the analyses and drafted the manuscript. JH participated in data interpretation and revised the manuscript. YX supervised the project and finalized the paper.

## Supplementary Material

Additional file 1 **NJ phylogeny built by using MEGA4**. See Legend of Figure 1. Click here for file

Additional file 2 Comparative study of CesA, CslA, CslC, CslD and CslF genes. Click here for file

Additional file 3 **The fasta format sequences of all Csl genes**. The description line is in the format: >ID|original ID|Csl family. Click here for file

Additional file 4 The original MSAs, the edited MSAs and the resulting phylogenetic trees. Click here for file
