# Supplementary figures and images for: The cellulose synthase superfamily in fully sequenced plants and algae
Source: BMC Plant Biol. 2009 Jul 31;9:99. doi: 10.1186/1471-2229-9-99 (PMC3091534; doi:10.1186/1471-2229-9-99)

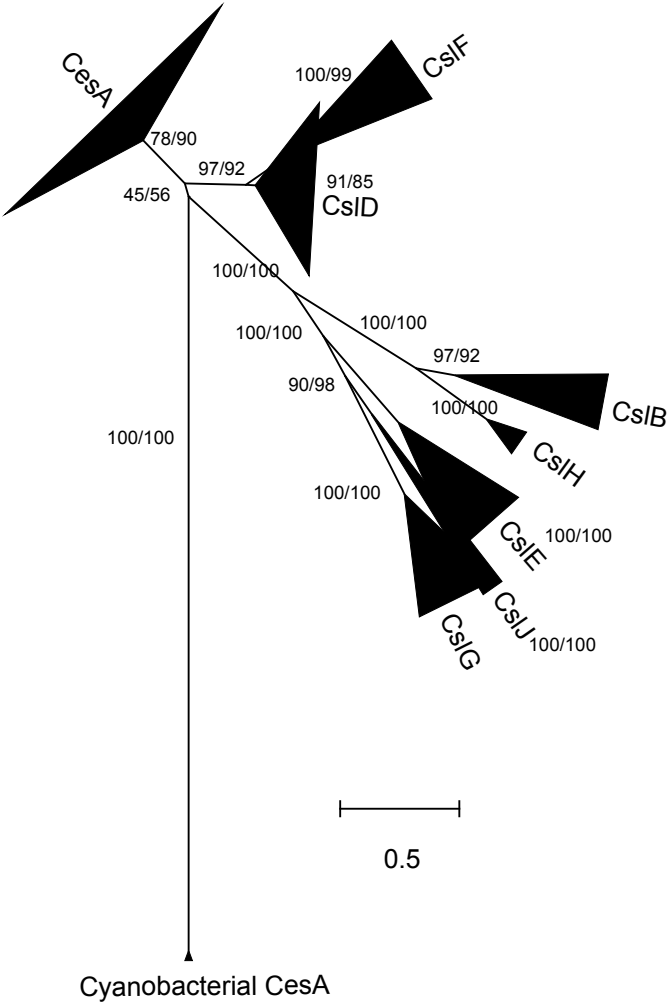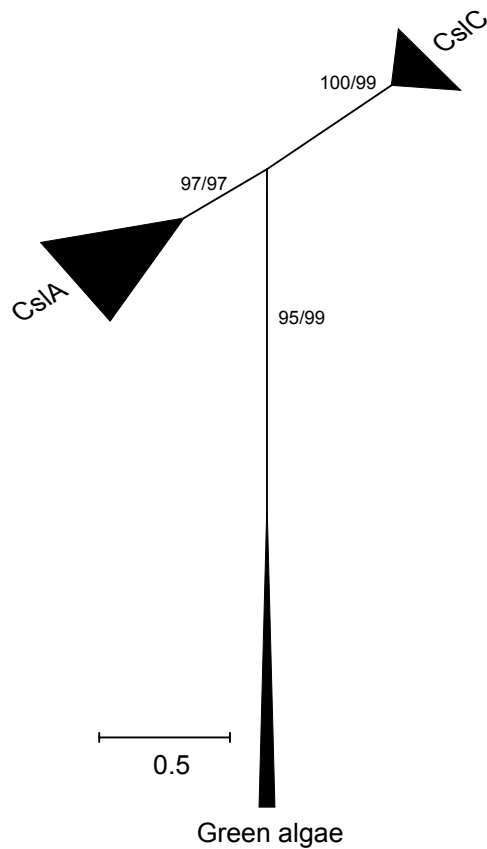

Supplement: Additional file 1 — NJ phylogeny built by using MEGA4. See Legend of Figure 1. [file 1471-2229-9-99-S1.pdf]

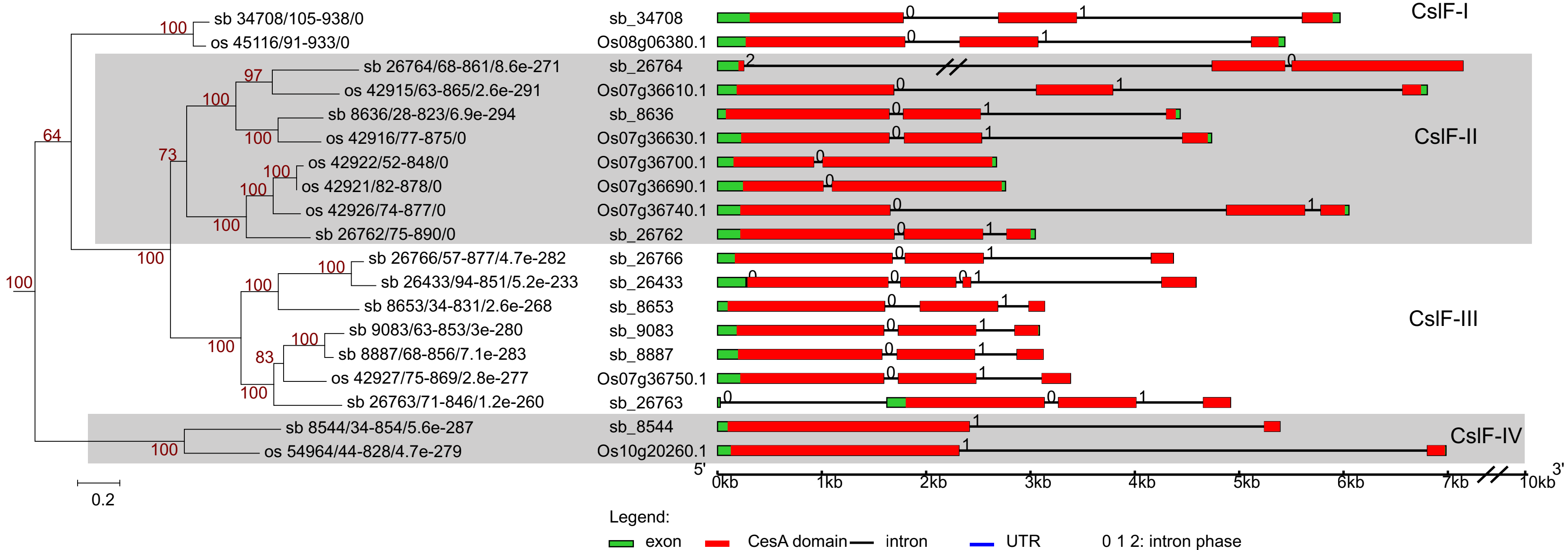

Supplement: Additional file 2 — Comparative study of CesA, CslA, CslC, CslD and CslF genes. [file 1471-2229-9-99-S2.zip › Fig3_cslF.domain.tre.pdf]

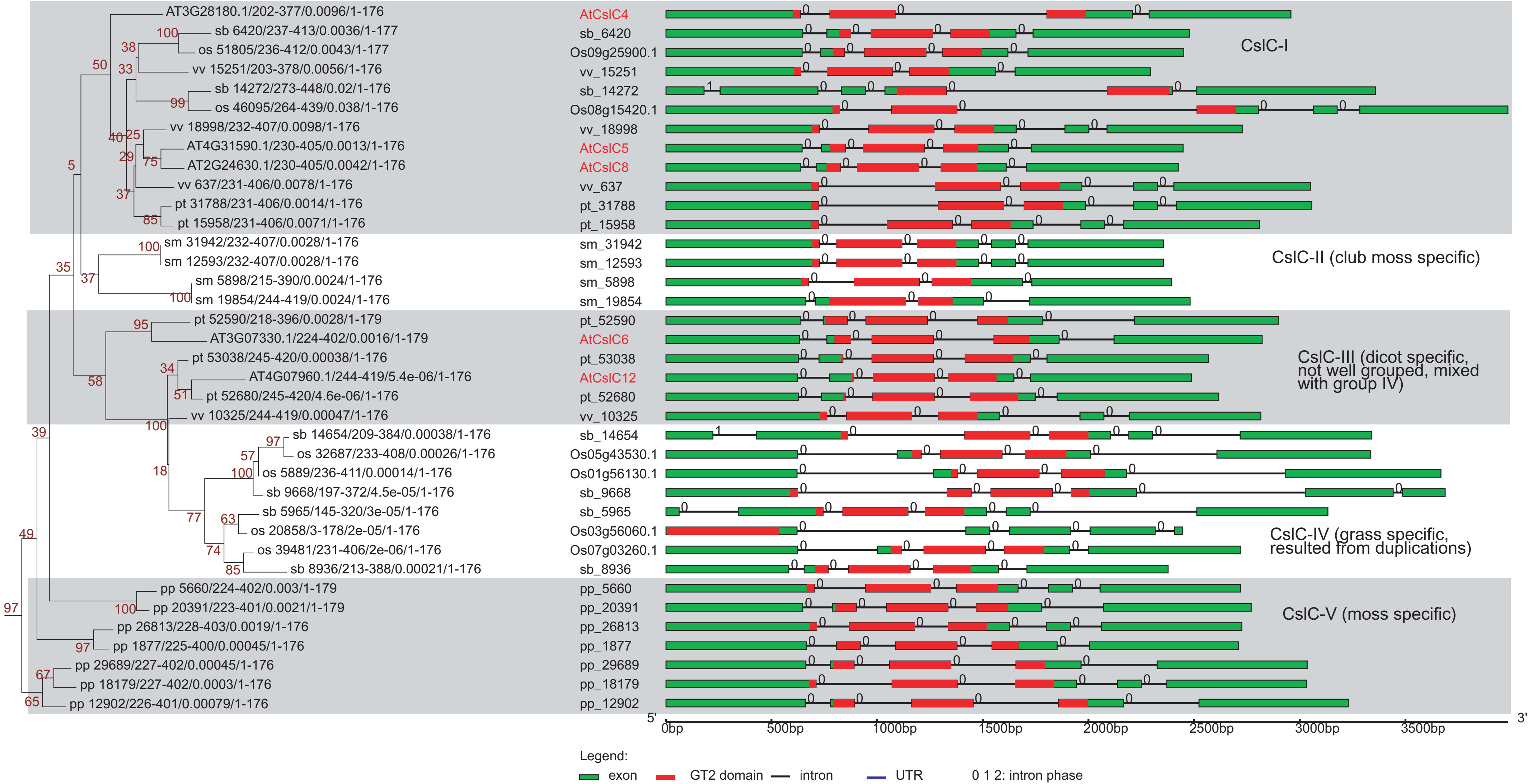

Supplement: Additional file 2 — Comparative study of CesA, CslA, CslC, CslD and CslF genes. [file 1471-2229-9-99-S2.zip › Fig5_cslC.domain.tre.pdf]

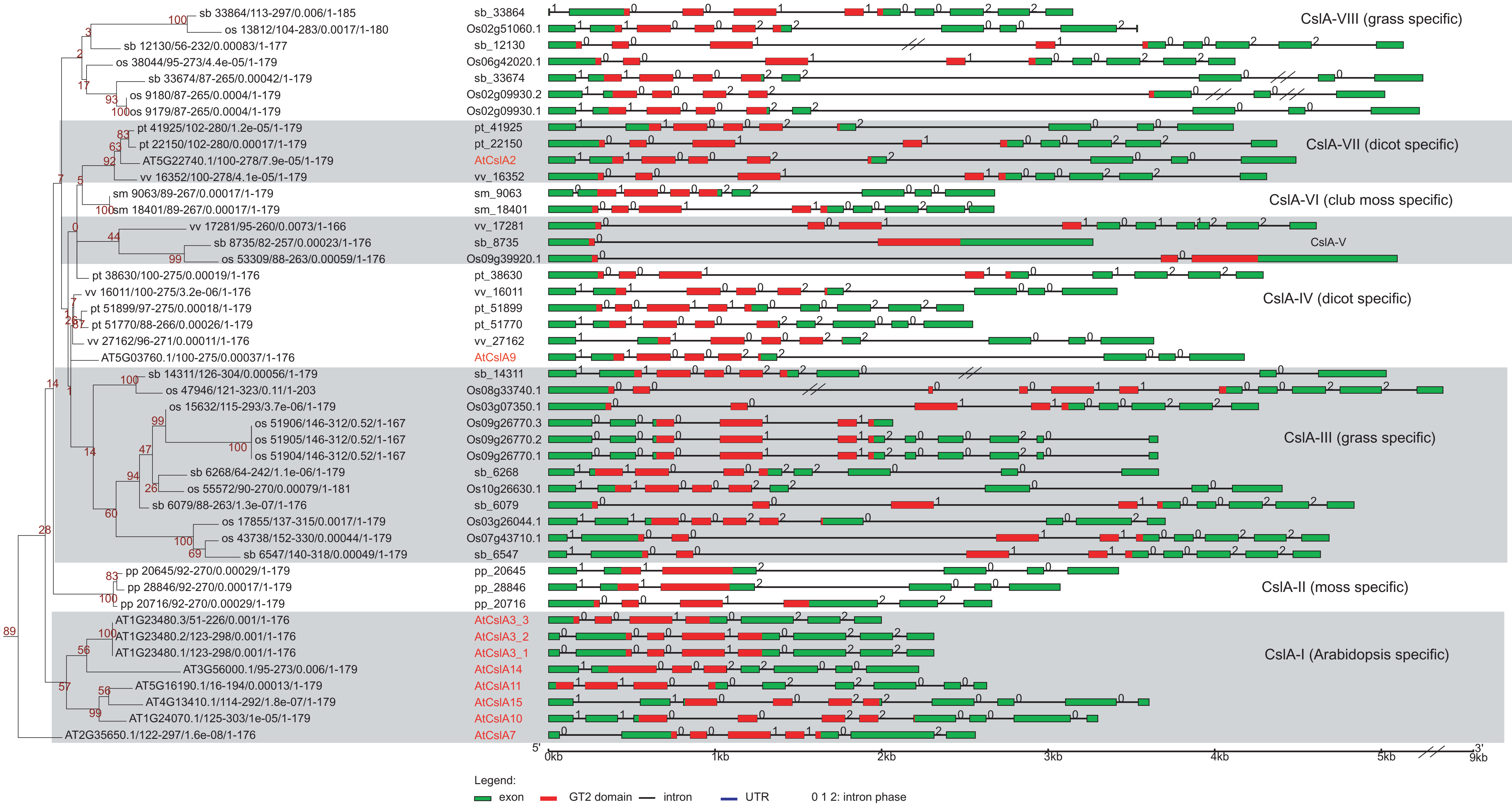

Supplement: Additional file 2 — Comparative study of CesA, CslA, CslC, CslD and CslF genes. [file 1471-2229-9-99-S2.zip › Fig4_cslA.domain.tre.pdf]

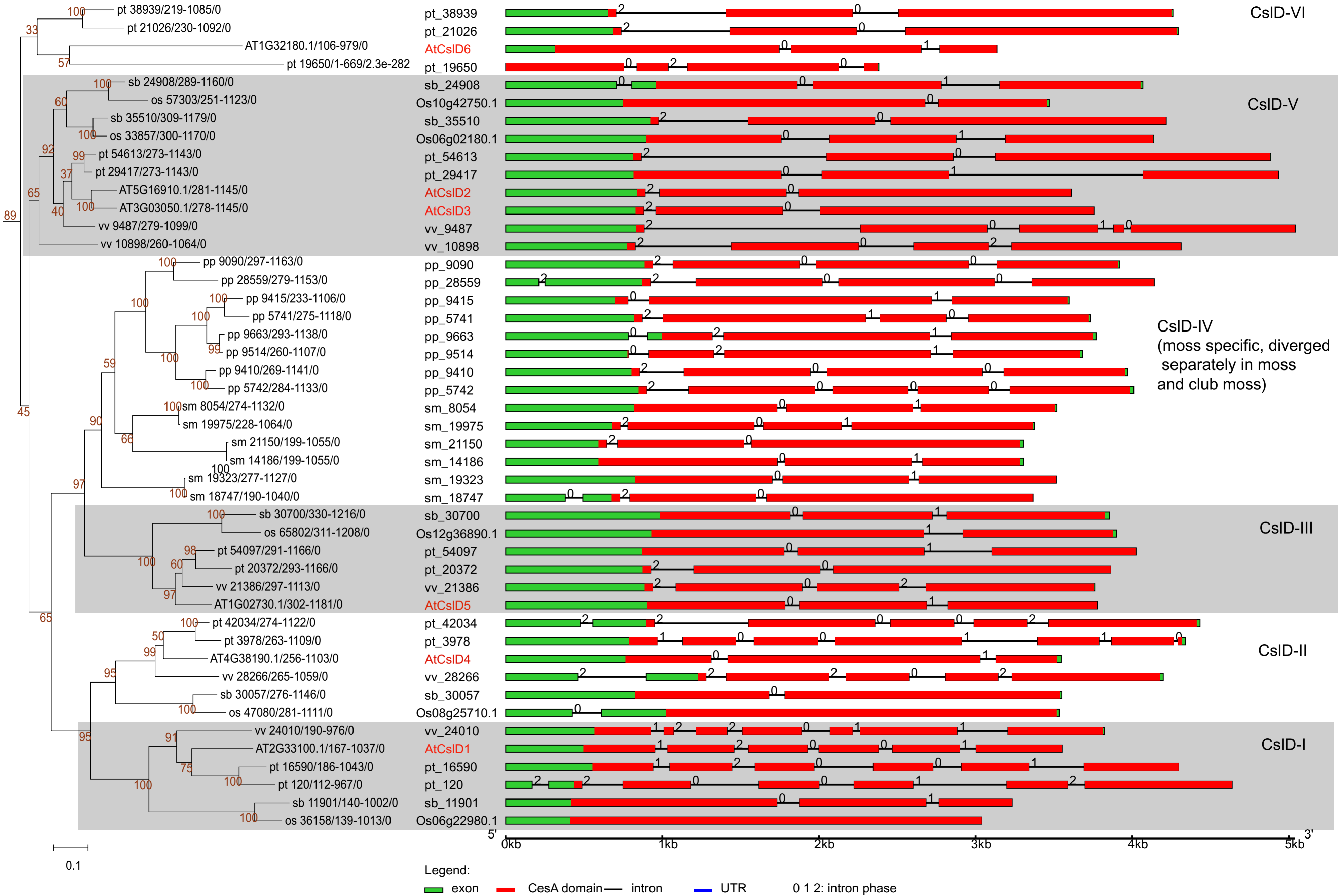

Supplement: Additional file 2 — Comparative study of CesA, CslA, CslC, CslD and CslF genes. [file 1471-2229-9-99-S2.zip › Fig2_cslD.domain.tre.pdf]

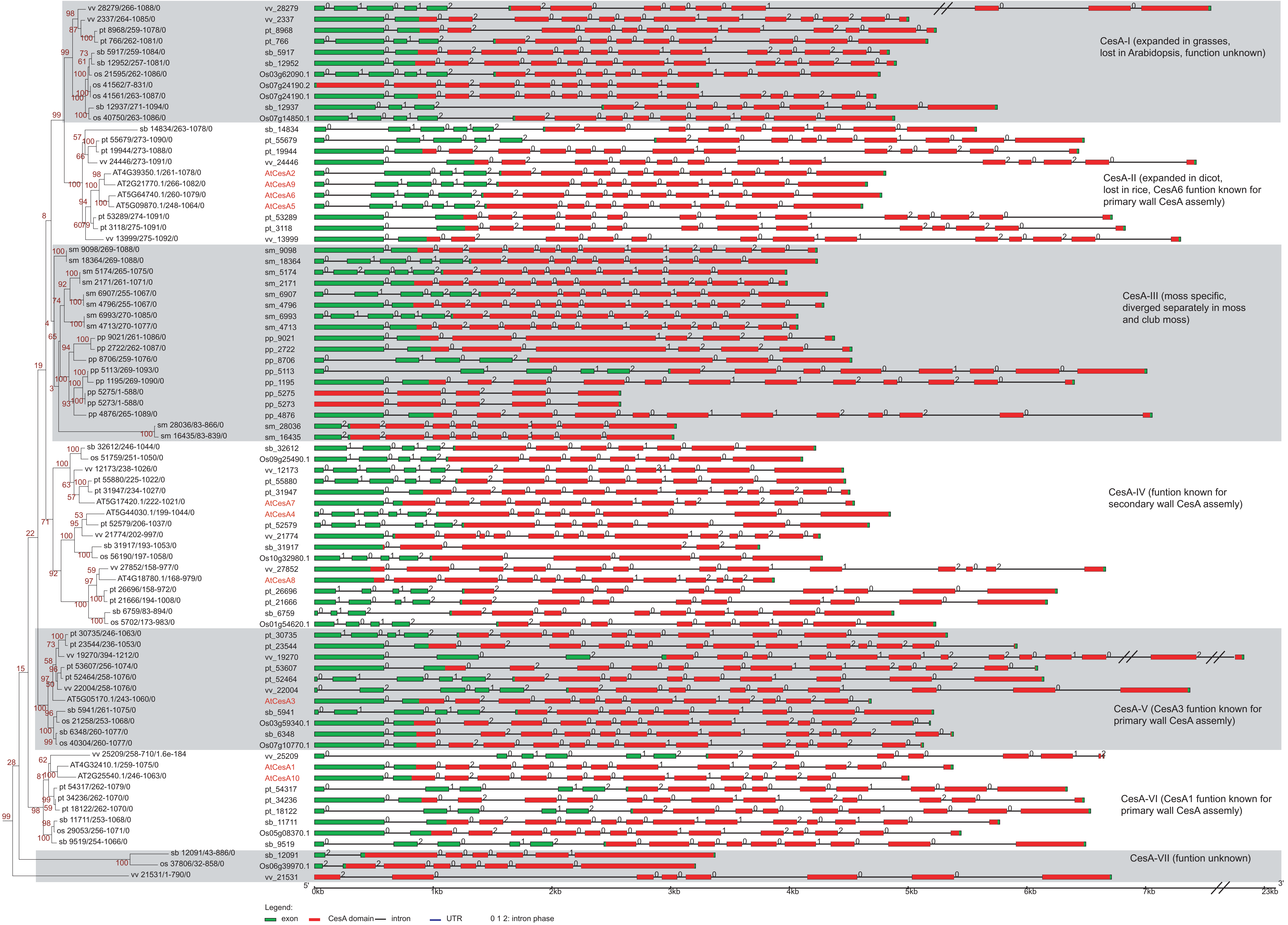

Supplement: Additional file 2 — Comparative study of CesA, CslA, CslC, CslD and CslF genes. [file 1471-2229-9-99-S2.zip › Fig1_cesA.domain.tre.pdf]

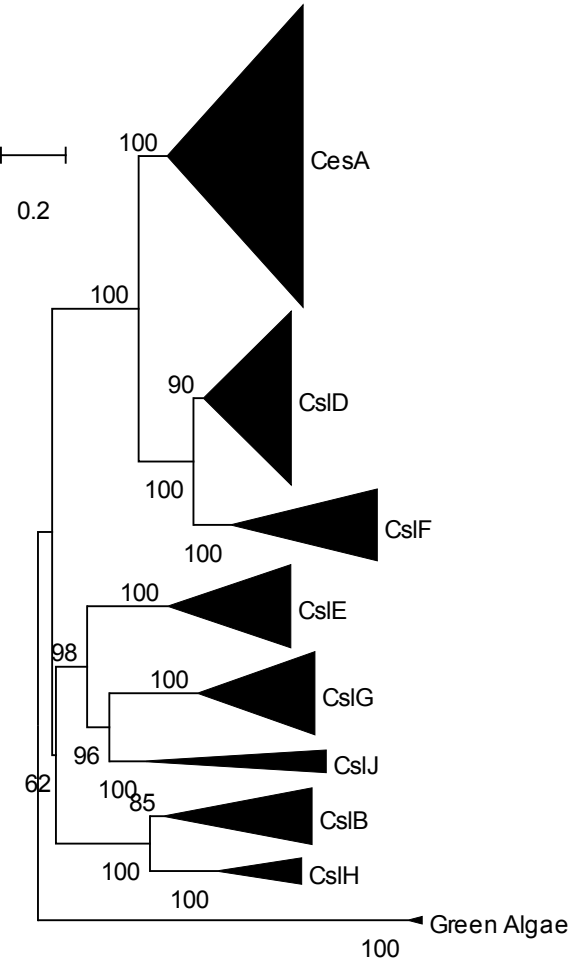

Supplement: Additional file 4 — The original MSAs, the edited MSAs and the resulting phylogenetic trees. [file 1471-2229-9-99-S4.zip › CesA.full.fa.e.pdf]

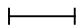

0.2

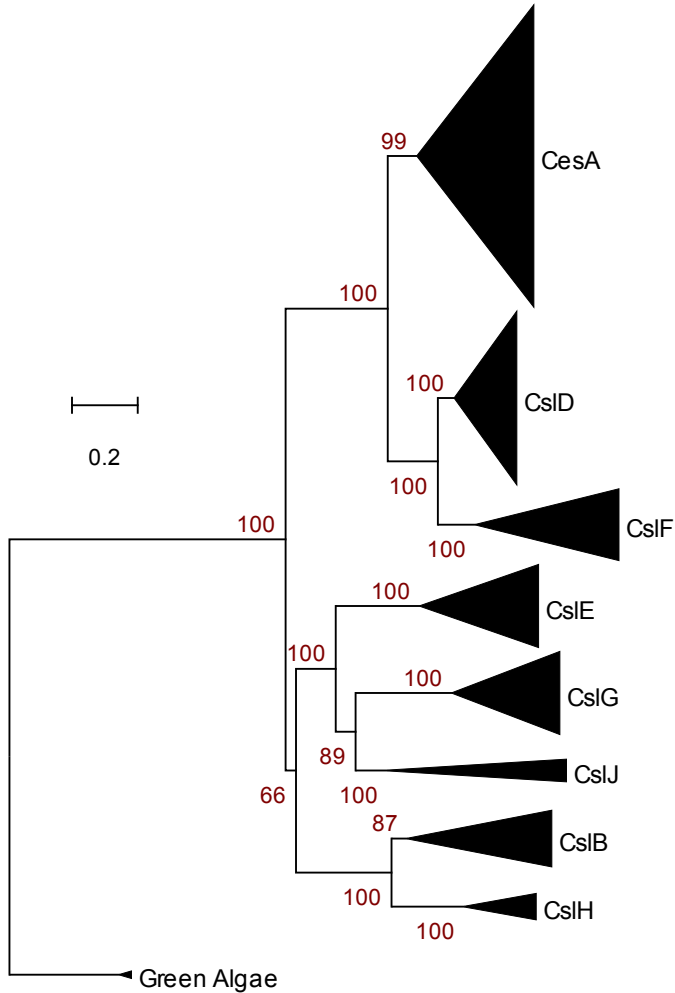

Supplement: Additional file 4 — The original MSAs, the edited MSAs and the resulting phylogenetic trees. [file 1471-2229-9-99-S4.zip › CesA.full.fa.e.2.pdf]

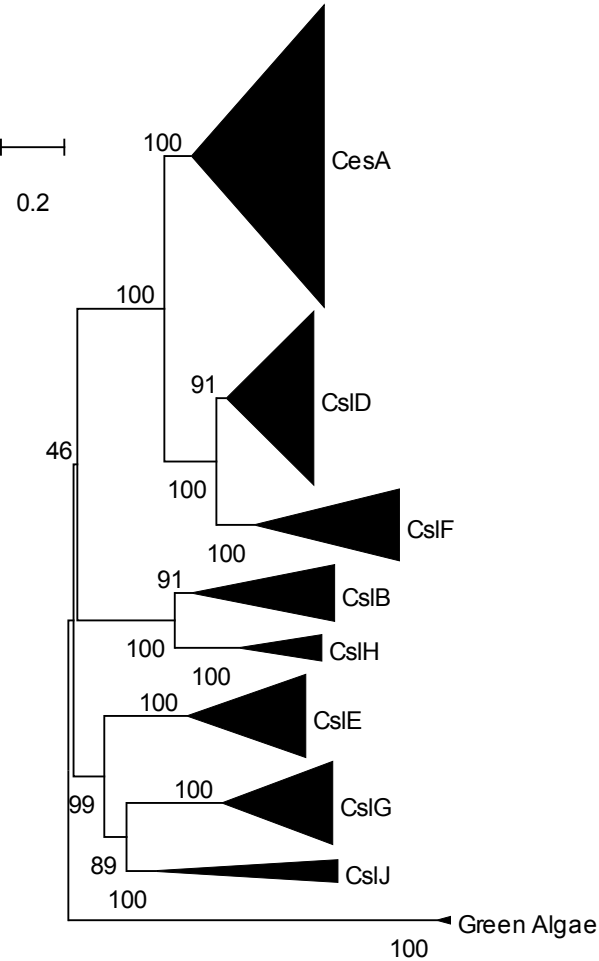

Supplement: Additional file 4 — The original MSAs, the edited MSAs and the resulting phylogenetic trees. [file 1471-2229-9-99-S4.zip › CesA.full.fa.l.pdf]

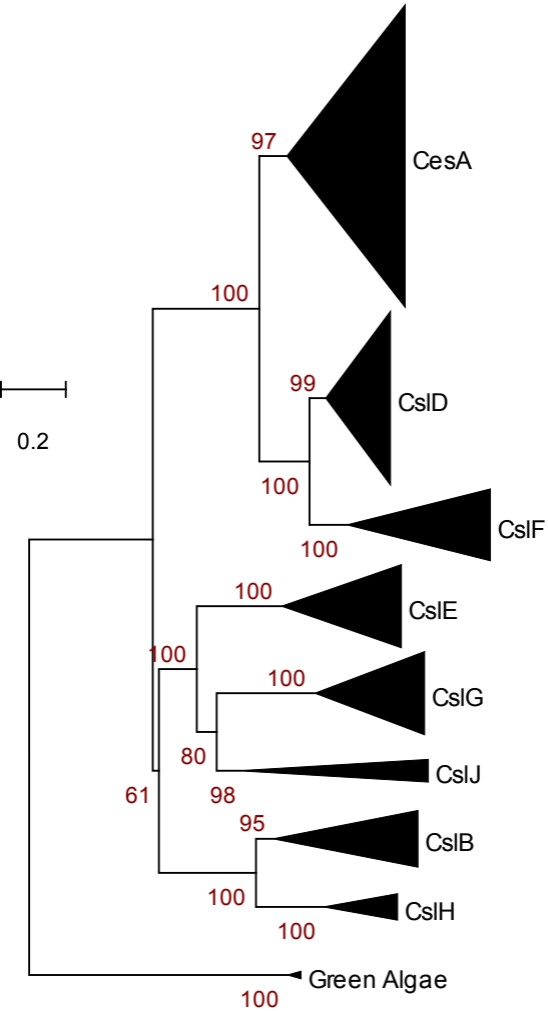

Supplement: Additional file 4 — The original MSAs, the edited MSAs and the resulting phylogenetic trees. [file 1471-2229-9-99-S4.zip › CesA.full.fa.l.2.pdf]
