# Supplementary material for: The cellulose synthase superfamily in fully sequenced plants and algae
Source: BMC Plant Biol. 2009 Jul 31;9:99. doi: 10.1186/1471-2229-9-99 (PMC3091534; doi:10.1186/1471-2229-9-99)
Supplement: Additional file 2 — Comparative study of CesA, CslA, CslC, CslD and CslF genes. [file 1471-2229-9-99-S2.zip › Csl_plants_suppl.pdf]

Comparative study of individual families

A) CesA family

We have classified the CesA family into seven subfamilies, as shown in Figure 1, all of which are supported by significant bootstrap values and have relatively conserved gene structures except the last one. We have observed the following:

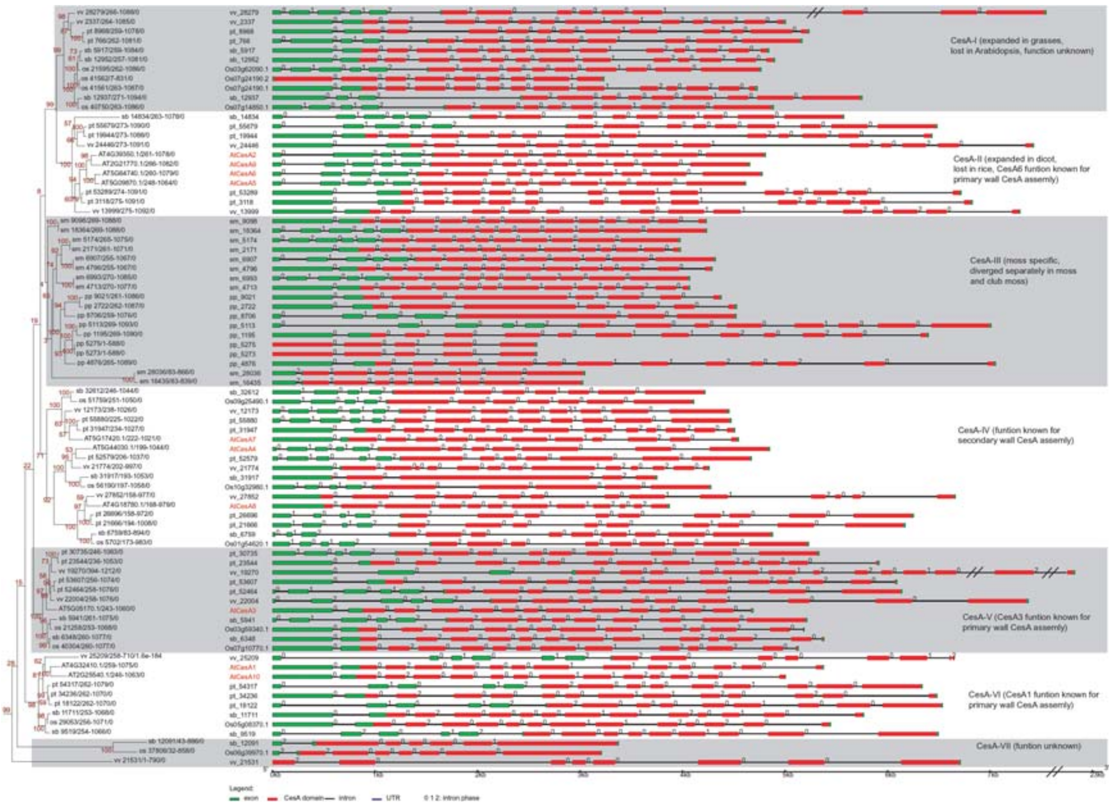

1) CesA genes have been well studied in Arabidopsis; however here our phylogenetic analysis found a CesA subfamily (group number I) does not have genes from this genome, suggesting it was lost in Arabidopsis. A Ka/Ks analysis indicates that genes in this group are under as strong purifying selection as other CesA subfamilies. A search against the EST database also revealed that these genes are highly expressed. So together they suggest that this CesA-I group is functionally active although it is absent in Arabidopsis.
2) The CesA-II group has four copies of Arabidopsis genes (AtCesA2,5,6,9), forming a monophyletic clade on the tree, and indicating that they could be possibly derived from a recent chromosomal polyploidy within the Arabidopsis lineage (Tang et al., 2008). Two very recent experimental reports (Desprez et al., 2007; Persson et al., 2007) have shown that these four genes are involved in the biosynthesis of cellulose in the primary cell wall, and are functionally redundant but have different expression profiles. Note that four copies of poplar (pt) genes and two copies of grape (vv) genes were found in this II group, but they were further put into two sub-subgroups, which may be derived from a more ancient chromosomal polyploidy (Tang et al., 2008). One Sorghum gene (sb_14834) was found in this group, but no rice homolog was found. For this particular grass gene, we found one EST match for it in the GenBank (gi|11679065) and the Ka/Ks ratio for it is 0.076, suggesting it is functionally active.

3) The CesA-III group consists of only moss (pp) and club moss (sm) genes. The phylogeny topology and the gene structures clearly suggest that they diverged separately within each species after the two species split.
4) The CesA-IV group consists of AtCesA4,7,8 genes, which are experimentally shown to be assembled into a complex for the cellulose biosynthesis in the plant secondary cell wall (Taylor et al., 2003). Our expanded dataset confirms Djerbi et al's finding (Djerbi et al., 2005), suggesting that CesA genes responsible for the secondary wall biosynthesis are likely to share a recent common ancestor.
5) The CesA-V and VI includes AtCesA3 and AtCesA1,10 respectively, and together with the CesA group II, are involved in the biosynthesis of cellulose in the primary cell wall (Desprez et al., 2007; Persson et al., 2007). According to the phylogeny given in Figure 1, these three groups of CesA, together with other CesA groups, diverged early in the evolution, suggesting that the biosynthesis of cellulose in the primary cell wall was much more anciently originated than that of secondary cell wall. Unlike group IV, we see multiple copies of CesA-V and VI genes in each of the five genomes, possibly a result of several rounds of chromosomal polyploidy (Tang et al., 2008).
6) Note in Table 2 poplar has roughly double of the number of CesA genes than that of other four flowering plants. This is reflected in the phylogeny in Figure 1: there are usually two or more copies of genes from poplar orthologous to one single gene from the other four angiosperm genomes on the tree. This happens in CslD family too (see Table 2). The reason is very likely due to a chromosomal polyploidy specific to *populus* (Tang et al., 2008).
7) The CesA-VII is marginal in the tree, including three genes. The Ka/Ks ratio test indicates that these genes are under less selection constraints (the Ka/Ks ratio, sb_12091: 0.3961, os_37806: 0.2246, vv_21531: 0.1541) comparing to the family median (0.04). An ESTs database search only found ESTs hits for vv_2153.

B) CslD family

Among all the Csl families, CslD is the phylogenetically closest to CesA family, but genes of the two families have very distinct gene structures, e.g. CslD genes have much less introns (2-3) than CesA genes have (~10). We have classified the CslD family into six subfamilies, each of which forms a well grouped monophyletic cluster, as shown in Figure 2. The CslD subfamilies I through III each has one copy gene from each of the 5 angiosperms except that poplar has two copies, which has also been observed in the CesA family (see above). In contrast, the group V has two copies of genes from each of the five angiosperms, probably caused by the chromosomal polyploidy (Tang et al., 2008). While it remains unknown as why plants keep multiple very similar copies of genes and how they work together to play their function, our Ka/Ks ratio analysis and EST expression data analysis suggest that they are all under strong selection constrains and should be functionally active. CslD-IV is a moss specific group, and as in CesA-III, the pp and sm genes were diverged separately by duplication after the speciation. Note that as in CesA family, pp genes and sm genes stay in a monophyletic group, suggesting they share a common origin. The last group VI is a small group including three poplar genes and AtCslD6, but note this grouping is marginal with weak support value and we didn't find EST homologs for the three pt genes in the GenBank.

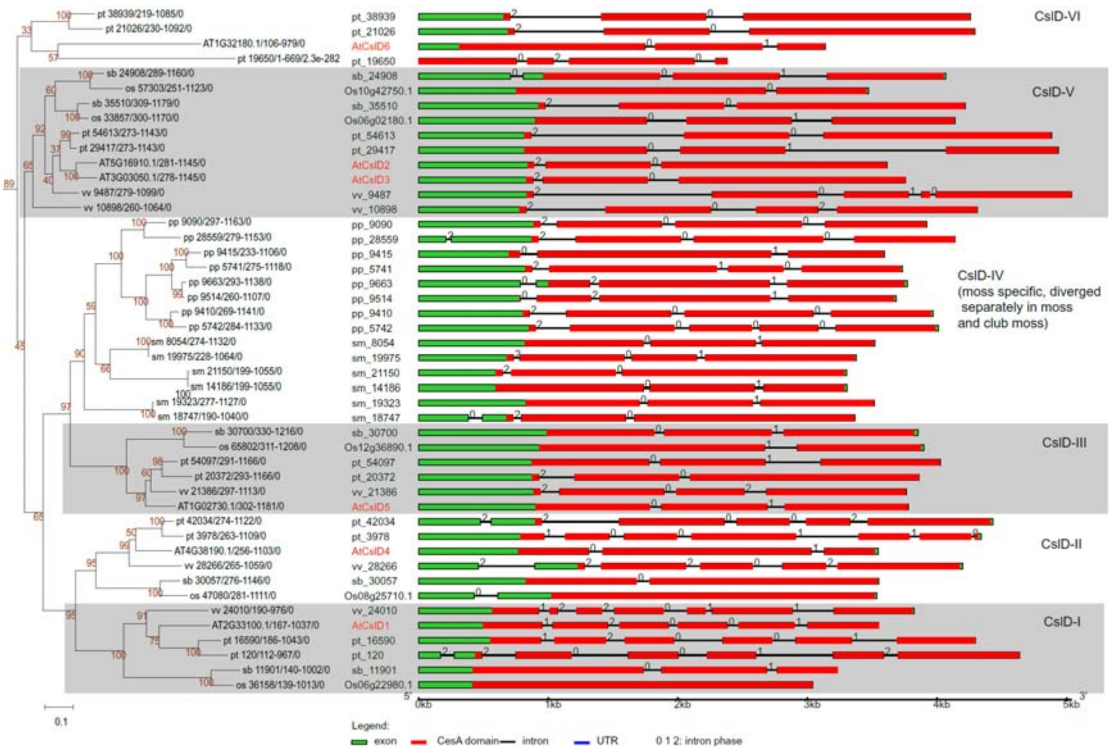

C) CslF family

We showed the phylogeny and the gene structures of CslF family in Figure 3. This family could be classified into four sub-families, and according to the phylogeny these sub-families diverged before rice and sorghum split. But note the group II is enriched with rice genes, while the group III is enriched with sorghum genes.

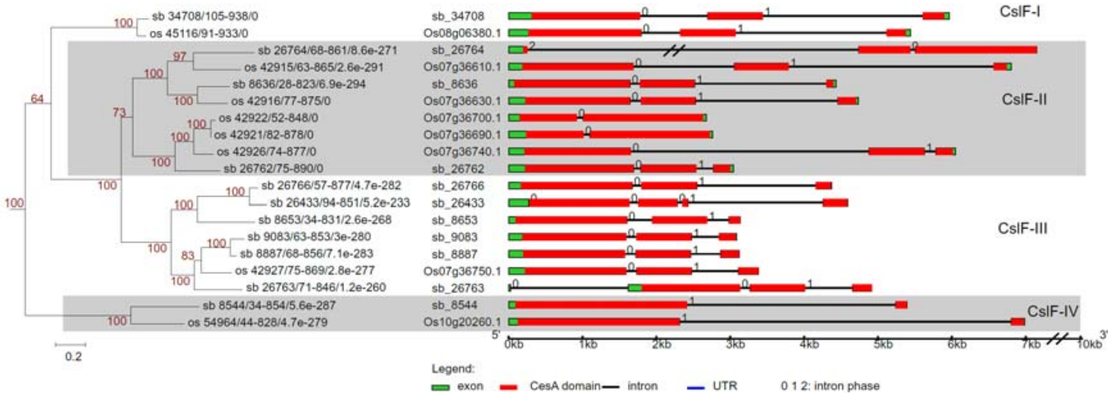

D) CslA family

As shown in Table 2, Arabidopsis, rice and sorghum have 8, 10 and 8 CslA genes respectively, while poplar and grape have only five and four CslA genes, suggesting the different duplication and subsequent depletion patterns in different genomes. Recently two reports have built phylogenies for CslA family by using sequences from diverse land plants (Suzuki et al., 2006; Liepman et al., 2007). One of them proposed that CslA diversified separately in the monocots and eudicots (Liepman et al., 2007).

Having genes in the tree from seven fully sequenced land plant genomes, shown in Figure 4, we were able to classify this family into eight subfamilies; this grouping is also supported by our NJ

tree (not shown), although both phylogenies were only able to provide good bootstrap supports for some of the groups. Basically, they include grass specific groups (group III and VIII), moss specific groups (group II and VI), Arabidopsis specific group (group I), dicot specific groups (group IV and VII), and the last group V, which groups one grape gene, one sorghum gene and one rice gene. According to this classification, sex out of eight Arabidopsis CslA genes form the monophyletic group I, while AtCslA2 and AtCslA9 are clustered with other dicot groups respectively; and all these three At groups were experimentally shown to encode mannose synthases (Liepman et al., 2005). Notably, the Ka/Ks ratio test shows that the omega value for AtCslA3 (AT1G23480) is 0.9532, suggesting it's under almost no selection constraint and possible to be a pseudogene. In summary, this classification apparently confirms Liepman's proposition suggesting that the diversification of most CslA family members indeed happened after the split of dicot and monocot grasses (Liepman et al., 2007), and the diversification of moss CslA genes happened separately as well (Roberts and Bushoven, 2007). However, note that pp genes and sm genes do not stay close to each other as they did in CesA and CslD, probably due to the low phylogeny quality of this one.

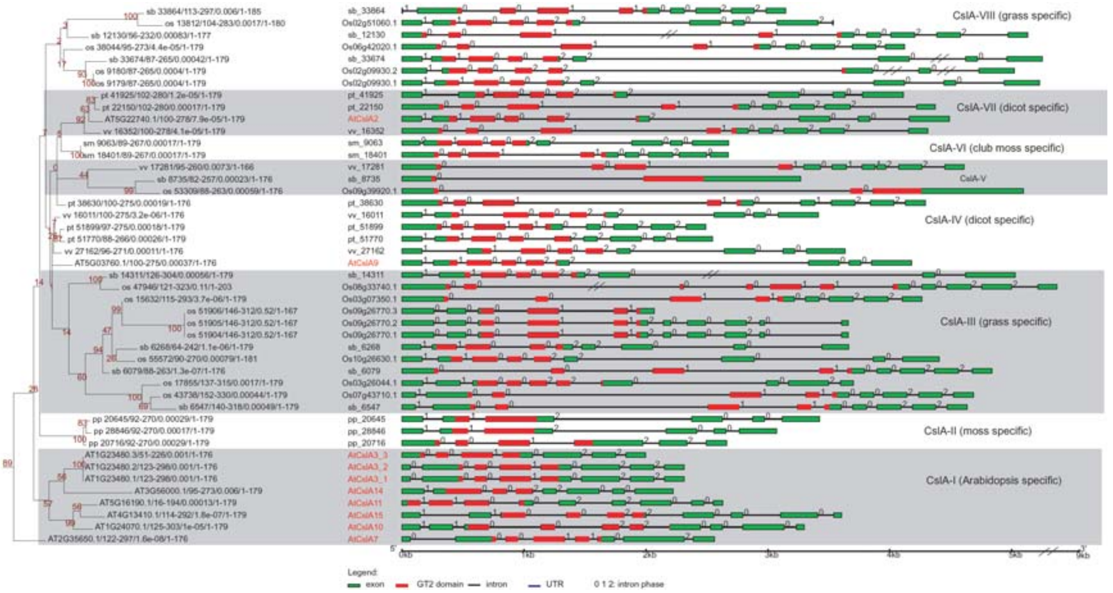

E) CslC family

Although very likely descended from a common ancestor as early as in the green algae (see above), comparing to CslA family, CslC is less diversified (see Figure 2), has less complex gene structure (e.g. less introns), and less complicated gene family phylogeny. And the last difference between the two families is that the 7 land genomes have the similar number of CslC genes (see Table 2). In Figure 5, we classified CslC family into five subfamilies. Note, as in CesA, CslD and CslA families, the mosses genes are always clustered into separate groups. Also note in fact, the group III and IV can be put into one single group, which means, there are only two large independent land plant CslC groups. The CslC-I includes only four grass genes, and they mixed with one grape gene. All the other grass genes form the group IV, which includes four subgroups, each with one rice gene and one sorghum gene, indicating they are derived from recent duplications.

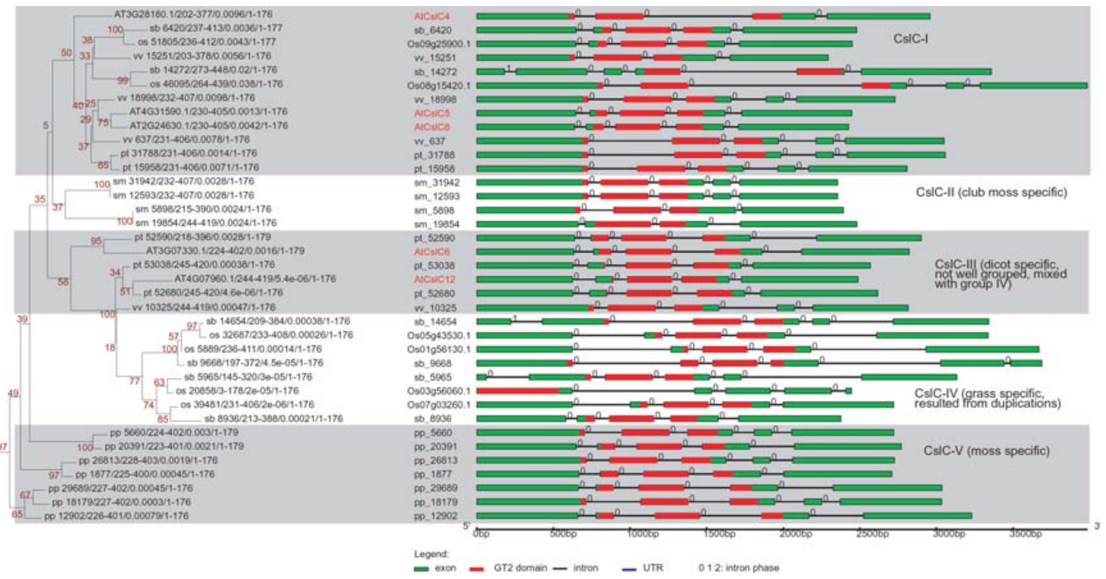

REFERENCE

Desprez T, Juraniec M, Crowell EF, Jouy H, Pochylova Z, Parcy F, Hofte H, Gonneau M, Vernhettes S (2007) Organization of cellulose synthase complexes involved in primary cell wall synthesis in Arabidopsis thaliana. Proc Natl Acad Sci U S A **104:** 15572-15577

Djerbi S, Lindskog M, Arvestad L, Sterky F, Teeri TT (2005) The genome sequence of black cottonwood (Populus trichocarpa) reveals 18 conserved cellulose synthase (CesA) genes. Planta **221:** 739-746

Liepman AH, Nairn CJ, Willats WG, Sorensen I, Roberts AW, Keegstra K (2007) Functional genomic analysis supports conservation of function among cellulose synthase-like a gene family members and suggests diverse roles of mannans in plants. Plant Physiol **143:** 1881-1893

Liepman AH, Wilkerson CG, Keegstra K (2005) Expression of cellulose synthase-like (Csl) genes in insect cells reveals that CslA family members encode mannan synthases. Proc Natl Acad Sci U S A **102:** 2221-2226

Persson S, Paredez A, Carroll A, Palsdottir H, Doblin M, Poindexter P, Khitrov N, Auer M, Somerville CR (2007) Genetic evidence for three unique components in primary cell-wall cellulose synthase complexes in Arabidopsis. Proc Natl Acad Sci U S A **104:** 15566-15571

Roberts AW, Bushoven JT (2007) The cellulose synthase (CESA) gene superfamily of the moss Physcomitrella patens. Plant Mol Biol **63:** 207-219

Suzuki S, Li L, Sun YH, Chiang VL (2006) The cellulose synthase gene superfamily and biochemical functions of xylem-specific cellulose synthase-like genes in Populus trichocarpa. Plant Physiol **142:** 1233-1245

Tang H, Bowers JE, Wang X, Ming R, Alam M, Paterson AH (2008) Synteny and collinearity in plant genomes. Science **320:** 486-488

Taylor NG, Howells RM, Huttly AK, Vickers K, Turner SR (2003) Interactions among three distinct CesA proteins essential for cellulose synthesis. Proc Natl Acad Sci U S A **100:** 1450-1455
